# Electro-Therapeutics

**Published:** 1894-01

**Authors:** 


Electro-Therapeutics of Neurasthenia. By W. F. Robinson, M. D. Price, paper cover, 25 cents; cloth, 50 cents. George S. Davis, Publisher, Detroit, Michigan.

This is a carefully prepared little volume of 72 pages, treating two of the most important questions of the day, viz.: Electricity, and functional nervous disease, both of which demand further investigation; hence a work ot this kind will meet with much favor. H.

